# Spatial structure facilitates the accumulation and persistence of antibiotic‐resistant mutants in biofilms

**DOI:** 10.1111/eva.12728

**Published:** 2018-12-22

**Authors:** Michael T. France, Ana Cornea, Hanna Kehlet‐Delgado, Larry J. Forney

**Affiliations:** ^1^ Institute for Bioinformatics and Evolutionary Studies University of Idaho Moscow Idaho; ^2^ Department of Biological Sciences University of Idaho Moscow Idaho; ^3^ Present address: Institute for Genome Sciences, School of Medicine University of Maryland Baltimore Maryland; ^4^ Present address: School of Medicine University of Washington Seattle Washington; ^5^ Present address: Department of Microbiology Oregon State University Corvallis Oregon

**Keywords:** adaptation, antibiotic resistance, bacteria, biofilm, selection

## Abstract

The emergence and spread of antibiotic resistance in bacterial pathogens are a global crisis. Because many bacterial infections are caused by pathogens that reside in biofilms, we sought to investigate how biofilms influence the evolution of antibiotic resistance. We hypothesize that the inherent spatial structure of biofilms facilitates the accumulation and persistence of spontaneously evolved antibiotic‐resistant mutants. To test this, we tracked the frequency of mutants resistant to kanamycin and rifampicin in biofilm populations of *Escherichia coli* before, during, and after an antibiotic treatment regimen. Our results show that biofilms accumulate resistant mutants even in the absence of antibiotics. This resistance was found to be heritable and thus unlike the phenotypic plasticity of so‐called “persister cells” that have been shown to occur in biofilms. Upon exposure to an antibiotic, resistant mutants swept to high frequency. Following the conclusion of treatment, these resistant mutants remained at unexpectedly high frequencies in the biofilms for over 45 days. In contrast, when samples from kanamycin‐treated biofilms were used to found well‐mixed liquid cultures and propagated by serial transfer, the frequency of resistant cells dramatically decreased as they were outcompeted by sensitive clones. These observations suggest that the emergence of antibiotic resistance through spontaneous mutations in spatially structured biofilms may significantly contribute to the emergence and persistence of mutants that are resistant to antibiotics used to treat bacterial infections.

## 1 INTRODUCTION

The US Centers for Disease Control and Prevention estimates that a majority of bacterial infections are caused by pathogens residing in biofilms (Costerton, Stewart, & Greenberg, 1999; Lebeaux, Ghigo, & Beloin, 2014; Poterra, 1999; R D Wolcott & Ehrlich, 2008), which are assemblages of microbial cells held together by an extracellular matrix (Stoodley, Sauer, Davies, & Costerton, 2002). Yet studies on the evolution of antibiotic resistance have largely used serially passaged planktonic populations as their experimental system. This distinction is important because the environment of bacterial populations in biofilms differs dramatically from that of their planktonic counterparts. Biofilm populations also experience complex diffusional gradients of nutrients and waste products that result in a wide array of physiological states and growth rates (Stewart & Franklin, 2008). Moreover, individual cells within a biofilm are restricted in movement, which provides the population with spatial structure. Infections caused by pathogens that form biofilms have been shown to be more difficult to eradicate with antibiotics than their planktonic counterparts (Wolcott et al., 2010; Xu, McFeters, & Stewart, 2000), often requiring longer treatment regimens (Römling & Balsalobre, 2012). We suspect that these differences may affect the evolution of antibiotic resistance, making it all the more important that this process be better understood.

We posit that the spatial structure of microbial biofilms strongly influences the evolution of antibiotic resistance. The fitness of antibiotic‐resistant clones is often reduced relative to their antibiotic sensitive ancestors (Andersson & Hughes, 2010; Andersson & Levin, 1999). In unstructured populations, this fitness cost increases the probability that resistant clones will be lost from the population in the absence of positive selective pressures exerted by antibiotics. This is because unstructured bacterial populations experience global competition (Hibbing, Fuqua, Parsek, & Peterson, 2010) wherein each individual competes against the entire population for resources and their reproductive success depends on their fitness relative to that of their competitors. In contrast, the situation in biofilms is strikingly different because individual cells are fixed in space by the extracellular polymeric matrix that they themselves produce (Sutherland, 2001). Thus, each individual only competes against a small subset of the population that is in close physical proximity. This limits the spatial scale at which natural selection can operate and protracts selective sweeps, thereby allowing less fit variants to persist and even accumulate (Gordo & Campos, 2006; Habets, Czárán, Hoekstra, & de Visser, 2007; Perfeito, Pereira, Campos, & Gordo, 2008). On this basis, we supposed that growth within a biofilm might facilitate the evolution and persistence of antibiotic‐resistant mutants.

Researchers have long known that resistance to most antibiotics, including aminoglycosides (Shakil, Khan, Zarrilli, & Khan, 2008), rifampicin (Jin, 1988), polymyxins (Port, Vega, Nylander, & Caparon, 2014), fluoroquinolones (Wolfson & Hooper, 1989), and β‐lactams (Sun, Selmer, & Andersson, 2014), can be achieved through mutations in target genes. These mutations occur in the absence of antibiotic selection at rates that are dependent on the number and mutation rate of the responsible genes as well as the bacterial strain in question (Courvalin, 2008). However, these heritable mechanisms of resistance have seldom been considered in the context of the recalcitrance of biofilms to antibiotic therapy. This is perhaps because it is commonly held that mutations are rare and of little consequence over short periods of time. However, this is misleading because although per cell mutation rates may be low, the sizes of bacterial populations in biofilms can be quite large. As a result, mutants resistant to any particular antibiotic may be common within any given biofilm population. Furthermore, these mutations are likely to be clinically relevant since that they have been identified in whole‐genome sequencing data from infections caused by several biofilm‐forming pathogens including *Staphylococcus aureus *(Howden et al., 2011; Mwangi et al., 2007), *Mycobacterium tuberculosis *(Eldholm et al., 2014)*, Enterococcus faecalis *(Arias et al., 2011)*, Klebsiella pneumoniae *(López‐Camacho et al., 2014)*, Pseudomonas aeruginosa *(Haidar et al., 2017; Marvig et al., 2015; Sommer et al., 2016; Tsukayama et al., 2004)*, *and *Acinetobacter baumannii *(Liu, Zheng, Zhang, Shen, & Zhao, 2016)*.*

Resistance is not the only means by which bacteria within biofilms can survive antibiotic exposure. For example, previous studies have shown that biofilm populations harbor antibiotic tolerant subpopulations that emerge from the differential expression of toxin–antitoxin system genes (e.g., hipAB) (Lewis, 2007, 2010). These tolerant cells persist during antibiotic treatment in a reversible state of dormancy (Schumacher et al.., 2015). The tolerant phenotype does not result from genetic change and is therefore not passed on to any of the surviving individuals’ offspring. Another example is the biofilm matrix itself, which can slow diffusion of the antibiotic thereby creating zones of lowered antibiotic concentrations where sensitive cells can survive (Hoyle, Alcantara, & Costerton, 1992; Stewart, 1996). Additionally, diffusional gradients of nutrients and resources in a biofilm allow for cells in a wide range of physiological states, some of which may be more tolerant to certain antibiotics (Sternberg et al., 1999; Xu, Stewart, Xia, Mcfeters, & Huang, 1998). For example, β‐lactam antibiotics such as penicillins, cephalosporins, and carbapenems that inhibit bacterial cell wall synthesis may not be effective against the nongrowing or slowly growing cells found in the interior of a biofilm. None of these mechanisms can account for the emergence of heritable drug resistance that is the bane of physicians attempting to cure chronic infections caused by biofilms.

The goal of this study was to better understand the role of mutation and selection in the development of biofilm recalcitrance to antibiotics by tracking the frequency of antibiotic‐resistant mutants in biofilm populations of *Escherichia coli *K12 MG1655 prior to, during, and after treatment with either rifampicin or the aminoglycoside antibiotic kanamycin. We hypothesized that (a) prior to antibiotic treatment, biofilm populations would accumulate antibiotic‐resistant mutants; (b) when these populations were treated with antibiotics, the resistant mutants would increase in frequency; and (c) growth within a biofilm would facilitate the persistence of mutants following the cessation of treatment.

## 2 MATERIALS AND METHODS

### 2.1 Strains, media, and biofilm cultivation

*Escherichia coli *K12 MG1655 was grown in an M9 salt‐based minimal glucose (12.2 mM) media supplemented with Wolfe's vitamins and trace elements (Wolin, Wolin, & Wolfe, 1963). Agar plates were prepared by supplementing the medium with 1.5% agar and, when appropriate, 20 µg/ml of either kanamycin or rifampicin. All planktonic cultures were grown at 37°C with shaking at 185 rpm. The minimum inhibition concentration (MIC) of the ancestor was determined by plating 5 µl droplets (containing approximately 10^4 ^CFUs) onto agar plates containing 0, 1.5, 3.0, 4.5, 6, 10, 15, or 20 µg/ml of either rifampicin or kanamycin. Plates were incubated for 24 hr and then examined for the presence of growth. We found the MIC of *E. coli* K12 MG1655 to be <1.5 µg/ml for kanamycin and 4.5 µg/ml for rifampicin.

**Figure 1:**
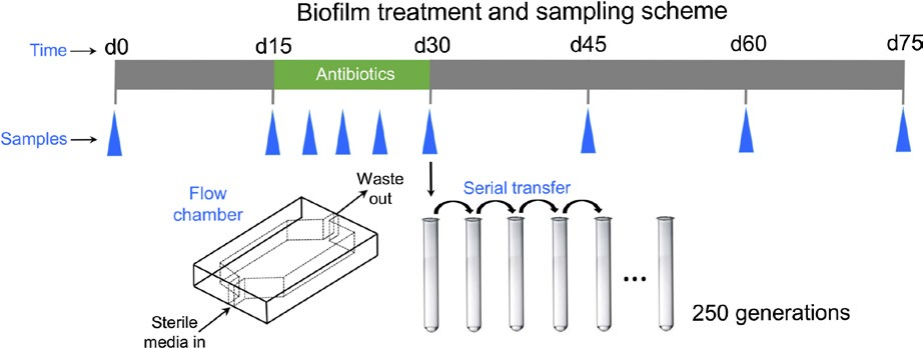
Schematic of the biofilm cultivation apparatus. Biofilms were grown for 75 days and treated with antibiotics from day 15 to day 30. Triplicate biofilms were destructively sampled at the time points denoted with blue triangles (18, 21, 25, and 30 days for kanamycin and 20, 25, and 30 days for rifampicin). Day 30 biofilm samples were used to inoculate a planktonic population that was serially passaged in the absence of antibiotics for 250 generations

Biofilms were cultivated at 25°C in custom acrylic flow cells with a glass substratum (total volume 10.4 ml) as described in Ponciano, La, Joyce, & Forney, 2009 (Ponciano et al., 2009). Media was supplied to the flow cells via a peristaltic pump fitted with syringe flow breakers to prevent upstream contamination and bubble traps to prevent the accumulation of bubbles (Figure 1). Flow cells were inoculated with 200 µl of an overnight culture using a needle and syringe. Following inoculation, a 24‐hr incubation period without flow was used to allow the bacteria time to adhere to the glass substrate. The flow of media was then commenced with a hydraulic retention time of 2 hr (5.4 ml/hr).

Biofilms were destructively sampled using a calcium/alginate entrapment technique. A liquid solution of 3% alginate was added to the flow cells over the course of 2 hr at a rate of 20 ml/hr, followed by a 1‐hr incubation period. Next, a 61.1 mM calcium chloride solution was added over the course of 2 hr at 20 ml/hr followed by another 1‐hr incubation. The calcium alginate mixture solidified providing a gel encased biofilm. The flow cells were then disassembled, and scalpel blades were used to remove three equally spaced, 1 cm by 2 cm horizontal sections from each biofilm. Biofilm sections were dissolved by placing them in a 0.85% saline (5 ml) solution and incubated at 37°C and shaking at 185 rpm for 2 hr.

The frequency of antibiotic‐resistant mutants in each biofilm section was then determined by diluting the sample in 0.85% saline and plating on selective and nonselective media in triplicate. All of the resistant genotypes identified in this study were capable of forming a colony on plates that contained 20 µg/ml of the respective drug, significantly higher than the ancestral MIC. This ensures that nonheritable persister strategies were not included in our experiments, because by definition they are not capable of growth in the presence of the antibiotic.

### 2.2 Treatment regimen

Biofilms were cultivated for 15 days in the absence of antibiotics and then treated with either kanamycin or rifampicin (30 µg/ml) for 15 days (Figure 1). After the treatment regimen, the biofilms were cultivated for a further 45 days in the absence of antibiotics. Triplicate biofilms were destructively sampled using the calcium alginate technique at the start of treatment (day 15), at several time points during treatment (kanamycin: days 18, 21, and 25; rifampicin: days 20 and 25), at the cessation of treatment (day 30), and at several time points following treatment (days 45, 60, and 75). Between the kanamycin and rifampicin treatment experiments, a total of 45 biofilms were used. After the initial 15 days of growth in the absence of antibiotics, *E. coli* K12 MG1655 was observed to have formed a robust biofilm that was attached to the glass substrate. The biofilms housed approximately 1,011 colony‐forming units from this point on, even during the 15‐day antibiotic treatment regime.

The triplicate biofilms harvested on the final day of treatment with either kanamycin or rifampicin (day 30) were used to inoculate planktonic cultures (5 µl of biofilm into 5 ml of media). These planktonic populations were then subcultured daily into fresh media (5 µl of culture into 5 ml of media). This passaging regime provided 10 generations of growth per day and was carried out for a total of 25 days (250 generations).

### 2.3 Determination of mutation rates and relative fitness

Mutation rates toward resistance to kanamycin and rifampicin were determined in triplicate for exponentially growing cultures as described in Rosche and Foster (2000) and analyzed using a maximum‐likelihood method (Hall, Ma, Liang, & Singh, 2009). For each replicate of the assay, 24 cultures were inoculated with <300 cells and grown until reaching stationary phase. Three of the 24 cultures were diluted and plated on nonselective plates to determine the average population size, and the remaining cultures were plated undiluted on selective media.

Mutation rates in stationary phase populations were determined by monitoring the increase in number of resistant mutants present during 5 days of stationary phase incubation by plating on selective and nonselective media every 24 hr. The rate of mutation was calculated as the slope of the increase in the abundance of antibiotic‐resistant mutants. There was no observed change in total population size during the 5 days.

The fitness of randomly selected antibiotic‐resistant mutants relative to the sensitive ancestor was determined as previously described (Lenski, Rose, Simpson, & Tadler, 1991). Cultures of antibiotic‐resistant mutants and the sensitive ancestor were grown separately overnight, and then, 2.5 µl of each was added to 5 ml of media. Mixtures of these clones were grown for 24 hr resulting in approximately 10 generations of growth. Final and initial densities of the resistant clone and the sensitive ancestor were determined by plating on selective and nonselective media. The relative fitness of each resistant clone was calculated as the ratio of the natural logarithm of the final over the initial population sizes.

### 2.4 Statistical analysis

All statistical analyses were performed using SAS 9.3 (SAS Institute, Cary, NC). The fraction of antibiotic‐resistant clones in the biofilm populations before, during, and after the treatment regime was analyzed using a factorial ANOVA with the log‐transformed fraction of resistant clones as the response variable and the antibiotic (either rifampicin or kanamycin), time point of the experiment, and their interaction as response variables. Post hoc comparisons of the fraction of resistance clones at various time points were used to test four hypotheses: that antibiotic‐resistant mutants accumulate in biofilms in the absence of antibiotics (day 0 vs. day 15), that these mutants sweep to high frequency during treatment (day 15 vs. day 30), that the evolved high proportion of resistant mutants can persist in the absence of selection in spatially structured environments (day 30 vs. days 45,60, and 75), and that growth within a biofilm was necessary for the persistence of the evolved resistance (day 75 vs. final abundance in planktonic). To correct for multiple comparisons, the Benjamini and Hochberg procedure was applied to the twelve comparisons using a false discovery rate of 0.05. Differences in the rate of mutation toward resistance to kanamycin and rifampicin and the relative fitness of resistant clones were determined using an unpaired two‐sample *t* test. Figures were prepared using R 2.1.4 (Team, 2013) and the plotrix package (Lemon, 2006). All of the data, SAS, and R code for this study can be found in the Supporting Information Appendix S1.

## 3 RESULTS

### 3.1 Populations within biofilms accumulated antibiotic‐resistant mutants in the absence of antibiotics

**Figure 2:**
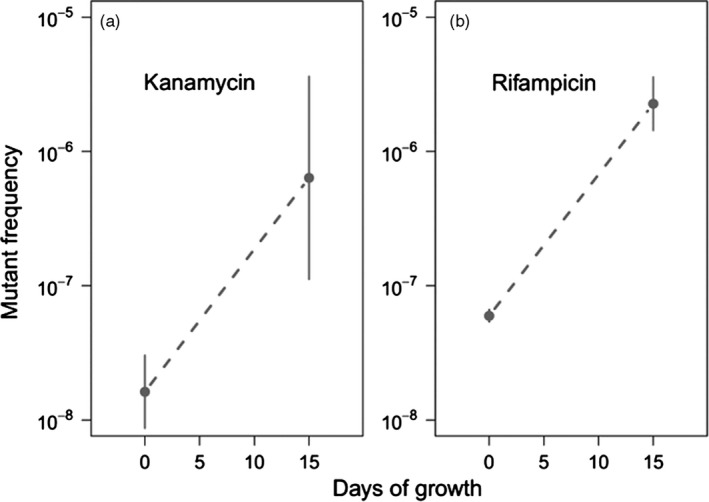
Log base ten‐transformed frequency of mutants resistant to either (a) kanamycin or (b) rifampicin after the first 15 days of growth as compared to those in the inocula (kanamycin, *p* = 0.0028; rifampicin *p* = 0.0012). Error bars represent 95% confidence intervals

We first tested whether antibiotic‐resistant mutants accumulated in biofilms prior to antibiotic treatment by comparing the frequency of rifampicin‐ and kanamycin‐resistant mutants in the initial inoculum to that of 15‐day‐old antibiotic‐naïve biofilm populations. The results showed that both kanamycin‐ and rifampicin‐resistant mutants were present at low frequency in the inoculum and increased in frequency during a 15‐day growth period in the absence of antibiotics (Figure 2a,b). Kanamycin‐resistant mutants increased from 2.3 × 10^−8^ in the inoculum to 1.1 × 10^−6^ in the 15‐day‐old biofilms—a 46‐fold increase (post hoc comparison; *t* = 3.6, *p* < 0.001). Similarly, rifampicin‐resistant mutants increased from 5.8 × 10^−8^ in the inoculum to 2.5 × 10^−6^ during the 15 days of biofilm growth—a 42‐fold increase (post hoc comparison; *t* = 21.7, *p* < 0.001). Comparable results were obtained when either tetracycline, cycloserine, or chloramphenicol was used in such experiments (Supporting Information Figure S1).

**Figure 3:**
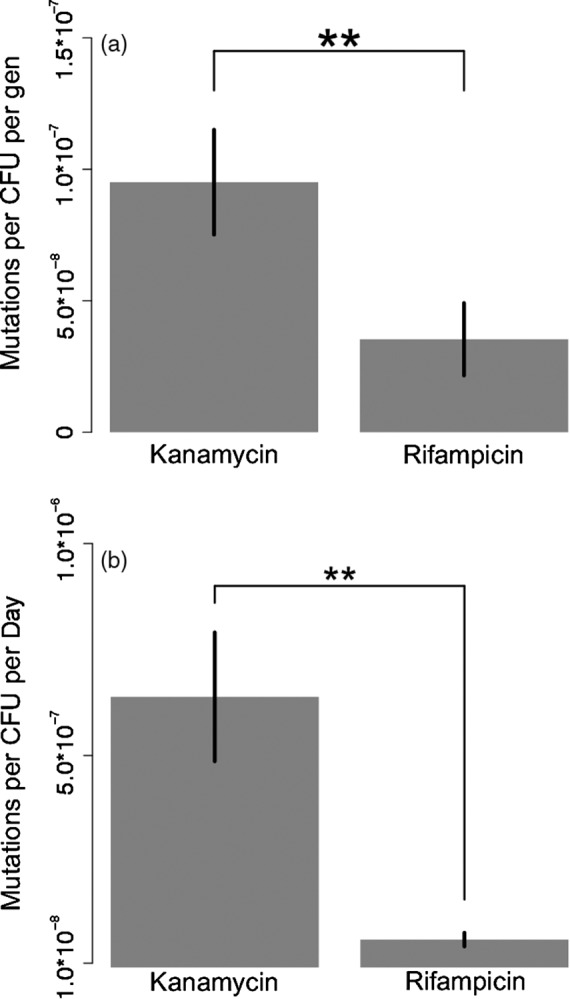
Comparison of the rate of mutation toward kanamycin and rifampicin resistance during exponential (a, *t* = 4.83, *p* = 0.0085) and stationary phases (b, *t* = 8.97, *p* < 0.001). *p < 0.05, **p < 0.01, ***p < 0.001, error bars represent 95% confidence intervals

The observed increase in frequency of resistant mutants prior to treatment (Figure 2a,b) could be driven by either (a) ongoing spontaneous mutations that generate new resistant genotypes or (b) selection that favors resistant genotypes in the absence of the antibiotics. If mutation alone accounts for the observed increase in the frequency of antibiotic‐resistant clones in the biofilms, then the rate of mutation (expressed per day) would need to match or exceed the per day rate of increase in the frequency of these mutants (7.0 × 10^−8^, per day for kanamycin‐resistant mutants and 1.6 × 10^−7^ per day for rifampicin‐resistant mutants). We measured the rate of mutation to kanamycin and rifampicin resistance in both exponentially growing and stationary phase populations and compared them to the observed increase in the frequencies of these mutants in the biofilm populations. In exponentially growing cultures, mutations that confer resistance to kanamycin and rifampicin occurred at rates of 9.5 × 10^−8^ and 3.5 × 10^−8^ mutations per colony‐forming unit (CFU) per generation, respectively (Figure 3a; *t* = 4.83, *p* = 0.0085). Based on these estimates, less than one generation of growth per day would be required to explain the increased frequency of kanamycin‐resistant mutants and approximately four and a half generations of growth to explain increased frequency of rifampicin‐resistant mutants. In stationary phase cultures, kanamycin‐resistant mutants emerged at a frequency of 6.4 × 10^−7^ mutations per CFU per day while rifampicin‐resistant mutants emerged at a frequency of 6.6 × 10^−8^ mutations per CFU per day (Figure 3b; *t* = 8.97, *p*<0.0001). These estimates for the per day mutation rate during stationary phase exceeded, in the case of kanamycin resistance, or approached, in the case of rifampicin resistance, the rate of increase in mutant frequencies observed in the biofilm populations prior to treatment.

**Figure 4:**
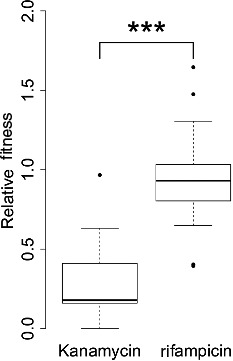
Comparison of the fitness of randomly selected resistant clones when grown in the absence of antibiotics relative to the sensitive ancestor, with kanamycin *n* = 12 and with rifampicin *n* = 18 (*t* = 5.79, *p* < 0.0001). *p < 0.05, **p < 0.01, ***p < 0.001, error bars represent 95% confidence intervals

If selection alone was responsible for the observed increase in the frequency of antibiotic‐resistant mutants prior to treatment, then the resistance mutations should not have large fitness costs in the absence of an antibiotic. To characterize the spectrum of fitness costs associated with the mutations that cause resistance to kanamycin and to rifampicin, we randomly selected 18 spontaneously resistant mutants and determined their fitness, in the absence of the antibiotic, relative to that of the sensitive ancestor. We found that mutations causing resistance to kanamycin were usually associated with a large fitness cost in this strain (Figure 4, average *s* = −0.72). Only one of the eighteen kanamycin‐resistant mutants exhibited a fitness similar to that of the ancestor and six had relative fitness values below the detection limit of the assay. In contrast, we found that the mutations that cause resistance to rifampicin are associated with only a small fitness cost and several resistant mutants were more fit than the ancestor even in the absence of rifampicin (Figure 4; average *s* = −0.056).

### 3.2 Antibiotic‐resistant mutants sweep during treatment but do not always reach fixation

**Figure 5:**
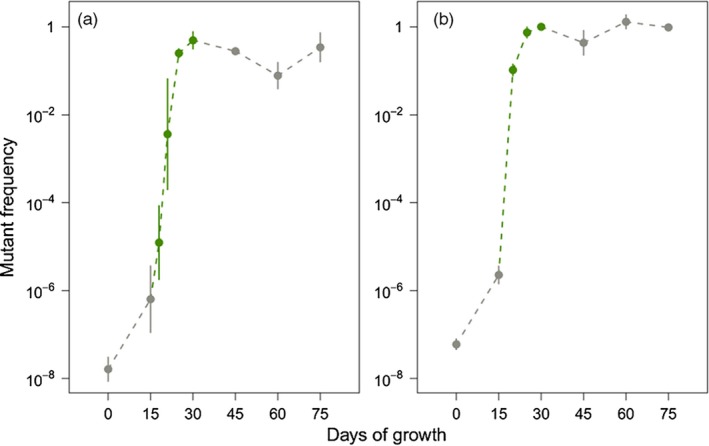
Log base ten‐transformed frequency of mutants resistant to either (a) kanamycin or (b) rifampicin before (gray), during (green), and after (gray) a 15‐day antibiotic treatment regime. Each point represents the average of three independent biofilms which were destructively sampled. For the experiments using kanamycin, a total of 24 biofilms were used, while 21 were used in the rifampicin study. Errors bars represent 95% confidence intervals

We expected that upon exposure to antibiotics, the preexisting resistant subpopulations would markedly increase in frequency but might not necessarily reach fixation in the population. To test this, we harvested kanamycin‐treated biofilms at 18, 21, 25, and 30 days and rifampicin‐treated biofilms at 20, 25, and 30 days (Figure 1) and determined the frequency of resistant mutants by plating on selective and nonselective media. As expected, the frequency of resistant mutants increased exponentially during the 15‐day treatment regime. However, the results obtained with the two antibiotics differed slightly. In the kanamycin‐treated biofilms, the frequency of resistant mutants increased from 1.1 × 10^−6^ at the start of treatment to 0.523 after 15 days of treatment (Figure 5a, *post hoc *comparison, *t* = 11.0, *p* < 0.001). Note that even after 15 days of treatment, kanamycin‐resistant mutants only accounted for roughly half of the total population. By comparison, the frequency of rifampicin‐resistant mutants increased from 2.5 × 10^−6^ resistant mutants per CFU in the naive biofilms to fixation at the end of treatment (Figure 5b, post hoc comparison, *t* = 60.9, *p* < 0.001).

### 3.3 Antibiotic‐resistant mutants can persist following the cessation of antibiotic treatment

Next, we determined whether these resistant mutants could persist following the cessation of treatment by monitoring the frequency of the resistant mutants in the post‐treatment biofilms for an additional 45 days in the absence of antibiotics. In both cases, the high abundance of resistant mutants did not substantially change following the removal of antibiotic selection. For kanamycin, the resistant clones comprised 52% of the population at the end of antibiotic treatment and still comprised 39% of the population after 45 days of antibiotic‐free cultivation (Figure 5a, *t* = −0.30, *p* = 0.77). Likewise, the rifampicin‐resistant mutants had fixed in the population by the end of antibiotic treatment, and this was unchanged after the removal of antibiotic selection (Figure 5b, *t* = −0.14, *p* = 0.89). These results demonstrate that the antibiotic resistance that developed during the treatment of biofilms persisted for extended periods of times even in the absence of antibiotics.

**Figure 6:**
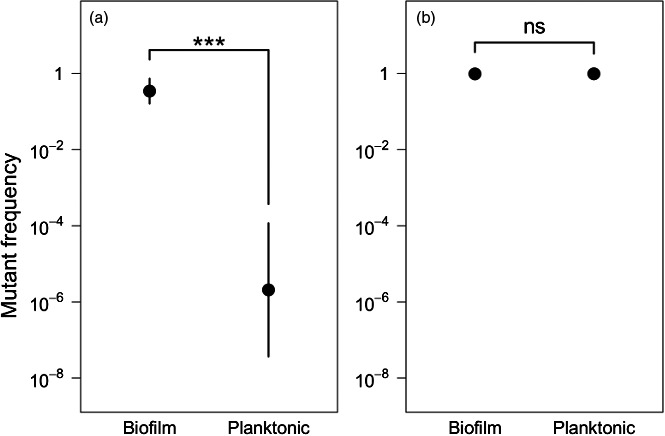
Comparison of the persistence of mutants resistant to either kanamycin (a, *t* = −9.77, *p* = 0.0003) or rifampicin (b, *t* = 0.02, *p* = 0.982) in the biofilm and planktonic populations. *p < 0.05, **p < 0.01, ***p < 0.001, error bars represent 95% confidence intervals

Finally, we determined whether growth within a biofilm was required for the persistence of the evolved high frequency of resistant mutants. Because well‐mixed populations experience global competition, we postulated that the frequency of the resistant mutants would decline when the populations were evolved in planktonic cultures. To test this, we founded planktonic populations using inocula from biofilm samples taken on the last day of treatment and serially passaged them for 250 generations in the absence of antibiotics. In the case of cultures founded from kanamycin‐treated biofilms, the high frequency of antibiotic‐resistant mutants steadily declined over time (Figure 6a) and after 250 generations the frequencies of resistant mutants in planktonic populations had returned to roughly that found in the inocula used to found the biofilms. This is in stark contrast to the persistence of kanamycin‐resistant mutants that was observed in the corresponding biofilm populations (Figure 6a, *t* = 9.77, *p* < 0.01). In comparison, rifampicin resistance persisted in both the biofilm and planktonic populations (Figure 6b, *t* = 0.02, *p* = 1.00). We think that the persistence of rifampicin‐resistant clones in planktonic cultures maybe due to the low fitness costs of rifampicin resistance, which would be expected to slow the sweep of rifampicin‐sensitive cells. These results indicate that growth within a biofilm facilitates the persistence of antibiotic‐resistant cells, even when resistance exacts a high cost on fitness.

## 4 DISCUSSION

The evolution of resistance to antibiotics used to eradicate bacterial pathogens is a significant factor that contributes to the morbidity and mortality caused by bacterial infections. As biomedical scientists pursue the development of new antibiotics to combat the growing prevalence of resistance, we must also develop a better understanding of the factors that influence the evolution of antibiotic resistance. In this study, we showed that residing within a biofilm facilitates the accumulation and persistence of antibiotic‐resistant mutants in bacterial populations. This is likely to play an important role in the overall evolution of resistance given that a majority of bacterial infections are caused by pathogens residing within biofilms.

In this study, we characterized the evolution of antibiotic resistance in biofilm populations of *E. coli* before, during, and after a 15‐day antibiotic treatment regime. We have shown that while the mutations that confer antibiotic resistance occur infrequently, they accumulate and are orders of magnitude more common in spatially structured biofilms than in well‐mixed cell suspensions, even in the absence of antibiotics. This was observed for several antibiotics with disparate mechanisms of action (Figure 2: kanamycin and rifampicin, Supporting Information Figure S1: chloramphenicol, tetracycline, and cycloserine). The resistant clones are pre‐adapted in terms of antibiotic resistance and as such are akin to the “seed banks” familiar to plant scientists in which preexisting seeds germinate when conditions are favorable (Leck, Parker, & Simpson, 1989).

The accumulation of resistant mutants in biofilms prior to treatment could be driven by continual spontaneous mutations that generate new resistant genotypes or by selection that favors resistant mutants in the absence of antibiotics. This distinction has important implications for the generality of our findings. If selection is responsible, then the observed increase in resistant mutants would hinge on whether specific antibiotic‐resistant mutants are able to outcompete the sensitive ancestor. However, if the increase is driven by spontaneous mutations, it only depends on whether resistance is obtainable via single‐step mutations. Here, we discuss our results in the context of mutation and selection and speculate about which evolutionary force is more likely to have driven the increase in antibiotic‐resistant mutants prior to treatment.

In their 2008 paper, Boles *et al* observed an accumulation of gentamycin‐resistant mutants in *Pseudomonas aeruginosa* biofilms which they hypothesized was driven by selective pressures within the biofilm (Boles & Singh, 2008). They pointed out that the complex diffusional gradients of nutrients and waste products present in biofilms are likely to create a diverse array of selective pressures. If any one such selective pressure favors the resistant mutants, it could account for the accumulation of resistant clones. We have shown that while the mutations responsible for rifampicin resistance have, on average, little effect on fitness (Reynolds, 2000), those responsible for kanamycin resistance have rather large fitness costs in the absence of the drug. Admittedly biofilm populations likely experience a diverse array of selective pressures, but it seems unlikely that there is a microenvironment within a biofilm that might favor kanamycin‐resistant mutants in the absence of the drug. Furthermore, biofilms have spatial structure and contain large subpopulations that are growing slowly (Sternberg et al., 1999). This limits competition to a local scale and impedes changes in allele frequency, a prerequisite for selective sweeps (Gordo & Campos, 2006; Habets et al., 2007; Perfeito et al.., 2008). Hence, the effect of selection on the frequency of antibiotic‐resistant clones is probably diminished in these slowly growing populations. This is consistent with our findings that indicate the rate of mutation toward resistance to both drugs was similar to the rate at which the frequency of resistant mutants increased in the biofilms prior to treatment. This implies that mutation alone might be able to account for this observation.

Due to technical limitations, we were not able to directly measure the rate of mutation in the biofilm populations. Instead, we used two different estimates for the mutation rate toward resistance: one derived from exponentially growing cultures and one from stationary phase cultures. It is likely that some combination of these two rates represent the average rate of mutation experienced by the biofilm populations. Using our estimates for the mutation rate during exponential growth, we have shown that relatively few generations of growth would be required per day to explain the accumulation of either kanamycin (<1 per day) and rifampicin (4.5 per day) resistance in biofilms. It seems likely that growth rate of the biofilms far exceeds these requirements given that they were provided with enough media to support twelve generations per day. Furthermore, even if the populations did not grow at all, which they most certainly did, our estimates for the rate of mutation in stationary phase cultures were also similar to the rate at which resistant mutants accumulated in the biofilms prior to treatment. We therefore conclude that spontaneous mutations alone are likely to be the major force responsible for the observed increase in abundance of rifampicin‐ and kanamycin‐resistant mutants prior to antibiotic treatment (see Supporting Information Appendix S1). To be clear, we are not suggesting that this accumulation of antibiotic‐resistant variants is driven by any foresight on the part of the bacteria, but instead that the population accumulates genetic diversity through a neutral process of spontaneous mutations. This explanation becomes even more attractive when you consider that several studies have hinted that mutation rates might even be elevated in biofilm populations (Boles & Singh, 2008; Driffield, Miller, Bostock, O'Neill, & Chopra, 2008; Ryder, Chopra, & O'Neill, 2012).

We also demonstrated that resistant mutants increased exponentially in frequency once the biofilms were treated with either kanamycin or rifampicin. This result was expected given that antibiotics select for any resistant genotypes in the population. However, somewhat unexpectedly, kanamycin‐resistant mutants did not fix in the population, and after 15 days of treatment, roughly half of the population was still sensitive to the antibiotic. These sensitive genotypes may have survived due to some combination of the previously mentioned phenotypic mechanisms of biofilm recalcitrance (antibiotic diffusion (Stewart, 1996), antibiotic action antagonism (Brown, Allison, & Gilbert, 1988), or the occurrence of persister cells (K Lewis, 2007)). In particular, aminoglycosides have been shown to not be as effective at killing under anaerobic conditions like that found in the interior of biofilm populations (Shakil et al., 2008). This problem is likely further compounded by the relatively low fitness of spontaneous kanamycin‐resistant mutants (Figure 4). In comparison, anaerobic conditions do not have as large of an effect on the activity of rifampicin and spontaneous rifampicin‐resistant mutants do not suffer the same fitness cost (Maggi, Pasqualu, Ballotta, & Sensi, 1966; Reynolds, 2000) and, accordingly, rifampicin‐resistant mutants fixed in biofilm populations. Whether or not resistant genotypes fix in the population has important implications for their persistence since, as the ancestral sensitive genotype declines in frequency, so does its chances of reemerging, following the cessation of treatment.

Perhaps, the most important result from this study is our demonstration that antibiotic‐resistant mutants persist at high frequency in biofilms even in the absence of antibiotics. We observed that after 45 days, the frequency of resistant mutants had not changed much from that found at the cessation of treatment. We suggest that this outcome probably derives from the same evolutionary forces responsible for the initial accumulation of resistant mutants. Biofilms are spatially structured and contained large subpopulations of slowly growing cells (Sternberg et al., 1999). These two factors limit the effectiveness of selection and facilitate the persistence of less fit variants such as kanamycin‐resistant mutants. This explanation is supported by our finding that the same kanamycin‐resistant mutants were rapidly expunged from well‐mixed planktonic populations. Moreover, the observed persistence of kanamycin‐resistant mutants in biofilm populations is consistent with clinical data from the treatment of recurrent biofilm infections and suggests that once an antibiotic has been used to treat a biofilm infection, its effectiveness may be permanently diminished (De Gelder et al., 2004). The persistence of large numbers of resistant mutants in the absence of antibiotics might also allow for the acquisition of additional compensatory mutations (Andersson & Hughes, 2010) that diminish the cost of resistance. We speculate that once the cost of resistance is ameliorated, the resistance mutants are more likely to persist even in well‐mixed populations, further facilitating the spread of antibiotic resistance (Andersson, 2003; Andersson & Hughes, 2010; Normak & Normak, 2002).

Above we have argued that the observed accumulation and persistence of antibiotic‐resistant mutants is not driven by positive selection, but instead by a relaxation of selection in biofilms that results from a combination of spatial structure and prolonged generation times. This argument is strengthened by our empirical estimates for the fitness cost associated with spontaneous kanamycin‐resistant mutants. However, our estimates for this fitness cost are derived from well‐mixed populations and not biofilms. It may be that the magnitude of the fitness cost associated with kanamycin resistance is diminished in the biofilms. We were unable to estimate the fitness cost of kanamycin resistance in biofilms because environmental heterogeneity makes it difficult to identify a single, meaningful value. A simple example of this is that that nutrient concentrations decrease with increasing depth in a biofilm due to reaction‐diffusion processes (Stewart & Franklin, 2008). If the magnitude of the specific fitness cost associated with kanamycin resistance is mitigated, or if the mutants actually have a fitness advantage in the absence of the drug under some subset of environmental conditions within a biofilm, this would also contribute to the accumulation and persistence of these variants.

One limitation of our study is that these experiments were done using a single strain of *E. coli*. Performing similar experiments with different combinations of species and antibiotics are needed to demonstrate the potential broad applicability of our results. However, we did conduct our experiments using two distinct antibiotics: one whose resistance mutations are associated with a large fitness cost and one whose resistance mutations are not. Previous studies have indicated the cost‐associated antibiotic resistance mutations can vary widely, although they are rarely as costly as the kanamycin resistance mutations characterized in this study (Melnyk, Wong, & Kassen, 2015). We speculate that this difference may result from the growth conditions used in this study. A previous study indicated that, for *E. coli*, the fitness cost of some resistance mutations are exacerbated when the strain is grown in minimal media, like that used in this study (Petersen, Aarestrup, & Olsen, 2009). Despite the stark differences in the fitness cost of rifampicin and kanamycin resistance mutations, our results for the two antibiotics are fairly similar. The primary differences were that rifampicin‐resistant variants fixed during treatment while kanamycin‐resistant variants did not and that kanamycin resistance declined in the unstructured populations. Overall, the results from this study demonstrate that biofilms play a critical role in the evolution of antibiotic resistance. Novel strategies must be developed for the treatment of persistent or recurrent biofilm infections that account for the inevitable rise and persistence of resistance in these populations.

## CONFLICT OF INTEREST

None declared.

## Data Availability

The data generated in this study and the code used to analyze it are available at: https://github.com/michaelfrance/evol_app_2018_biofilms.
