# Supplementary material for: Spatial structure facilitates the accumulation and persistence of antibiotic‐resistant mutants in biofilms
Source: Evol Appl. 2018 Dec 22;12(3):498–507. doi: 10.1111/eva.12728 (PMC6383844; doi:10.1111/eva.12728)

Supplemental Figure S1: Log base ten transformed frequency of mutants resistant to either chloramphenicol, kanamycin or tetracycline after the first 15 days of growth as compared to those in the inocula. Error bars represent 95% confidence intervals.


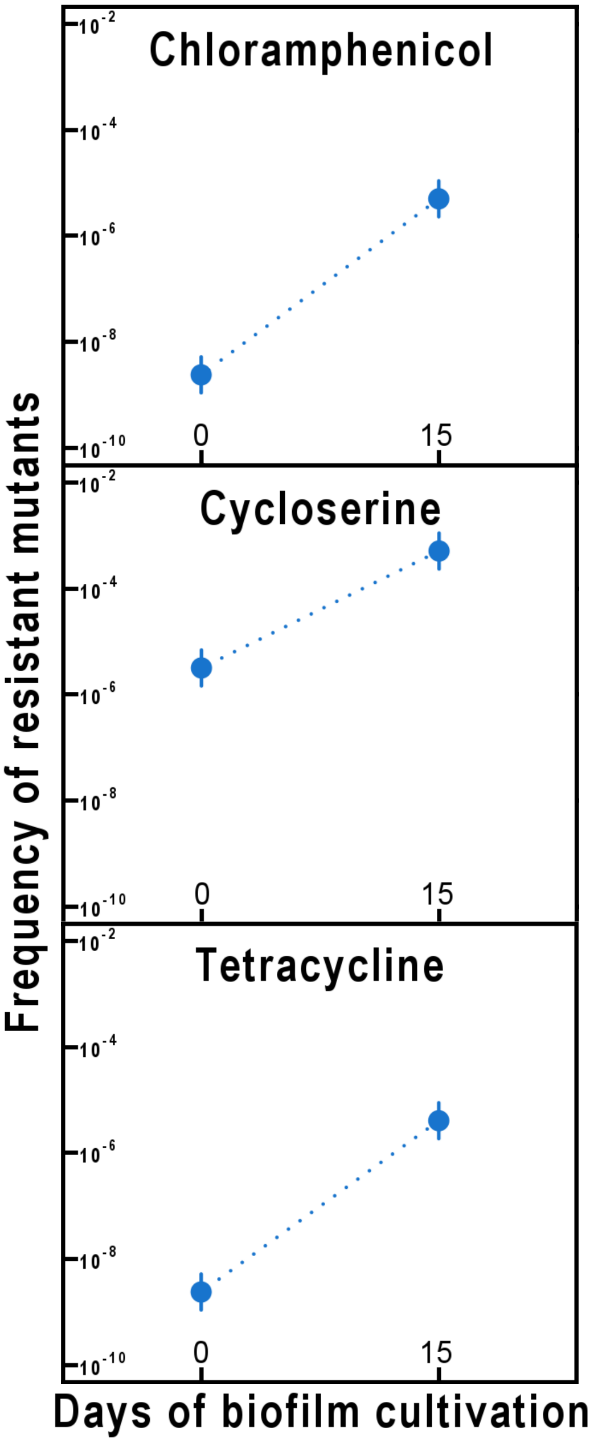

Supplement: Supplementary file 1 [file EVA-12-498-s001.docx]
