# Supplementary material for: Spatial structure facilitates the accumulation and persistence of antibiotic‐resistant mutants in biofilms
Source: Evol Appl. 2018 Dec 22;12(3):498–507. doi: 10.1111/eva.12728 (PMC6383844; doi:10.1111/eva.12728)
Supplement: Supplementary file 2 [file EVA-12-498-s002.docx]

# Supporting information

We showed that biofilm populations accumulated kanamycin and rifampicin resistant variants prior to antibiotic treatment. One possible explanation for this increase is that these resistant mutants were selected for in the biofilm despite the absence of the antibiotic. However, we argue that it is more likely that the increase was driven by a neutral process of spontaneous mutation. Here, we present simple calculations using our own empirical estimates of the mutation rates towards kanamycin and rifampicin resistance to demonstrate that it is plausible that spontaneous mutations drove the observed accumulation of resistant cells.

Two different mutation rates toward resistance to each antibiotic were considered, one for exponentially growing cells and one for stationary phase cells. It is likely that members of a biofilm population experience some combination of the two rates depending on their location in a biofilm. Cells near the top have first access to nutrients in the bulk medium and are therefore likely to resemble those in exponentially growing liquid cultures. Whereas cells near the bottom experience limited growth due to a lack of nutrients and are likely to more closely resemble cells in stationary phase liquid cultures. For this reason, we have calculated both the number of generations of exponential growth required and the number of hours in stationary phase that would be required to explain the observed accumulation of resistant clones. It is likely that some combination of these two estimates best describes the experimental data.

## Kanamycin

We observed that the frequency of kanamycin resistant (Kan^r^) mutants increased from 2.31 x 10^-8^ Kan^r^ mutants per CFU at the time of inoculation, to 1.07 x 10^-6^ Kan^r^ mutants per CFU after 15 days of antibiotic-free cultivation.

If we assume that the increase from day 0 to day 15 was linear, then Kan^r^ mutants increased in frequency at a rate of 6.98*10^-8^ new Kan^r^ mutants per CFU per day. In order for mutation alone to explain this increase, the mutation rate would need to exceed this value.

## *Analysis using mutation rate during exponential growth*

Rate of increase observed in biofilms: 6.98*10^-8^ new Kan^r^ mutants/CFU/day

Rate of mutation to kanamycin resistance: 9.48x10^-8^ new Kan^r^ mutants /CFU/gen

$$\frac{6.98*{10}^{-8}new kan. res. mutants per CFU per day}{9.48*{10}^{-8} new kan. res. mutants per CFU per gen}=0.74 gen per day$$

## *Analysis using mutation rate during stationary phase*

Rate of increase observed in biofilms: 6.98*10^-8^ new Kan^r^ mutants/CFU/day

Rate of mutation to kanamycin resistance: 6.38x10^-7^ new Kan^r^ mutants /CFU/day

$$\frac{6.98*{10}^{-8}new kan. res. mutants per CFU per day}{6.38*{10}^{-7} new kan. res. mutants per CFU per day}=0.11$$

Kan^r^ mutants increased in the biofilms at approximately 1/10 the rate of mutation during stationary phase.

## Rifampicin

We observed that the frequency of rifampicin resistant (Rif^r^) mutants increased from 5.81 x 10^-8^ Rif^r^ mutants per CFU at inoculation, to 2.46 x 10^-6^ Rif^r^ mutants per CFU after the 15 days of antibiotic-free cultivation.

If we assume that the increase from day 0 to day 15 was linear, Rif^r^ mutants increased in frequency at a rate of 1.60*10^-7^ new Rif^r^ mutants per CFU per day. In order for mutation alone to explain this increase, it would need to meet or exceed this rate.

## *Analysis using mutation rate during exponential growth*

Rate of increase observed in biofilms: 1.60*10^-7^ new Rif^r^ mutants/CFU/day

Rate of mutation to rifampicin resistance: 3.54x10^-8^ Rif^r^ mutants /CFU/gen

$$\frac{1.60*{10}^{-7}new rif. res. mutants per CFU per day}{3.54*{10}^{-8} new rif. res. mutants per CFU per gen}=4.52 gen per day$$

## *Analysis using mutation rate during stationary phase*

Rate of increase observed in biofilms: 1.60*10^-7^ new Rif^r^ mutants/CFU/day

Rate of mutation to kanamycin resistance: 2.74x10^-9^ new Rif^r^ mutants /CFU/day

$$\frac{1.60*{10}^{-7}new rif. res. mutants per CFU per day}{6.58*{10}^{-8} new rif. res. mutants per CFU per day}= 2.43$$

Rif^r^ mutants increased in the biofilms at approximately 2.5 times the rate of mutation during stationary phase.
